# Physiological, Biochemical, and Biophysical Characterization of the Lung-Lavaged Spontaneously-Breathing Rabbit as a Model for Respiratory Distress Syndrome

**DOI:** 10.1371/journal.pone.0169190

**Published:** 2017-01-06

**Authors:** Francesca Ricci, Chiara Catozzi, Xabier Murgia, Brenda Rosa, Davide Amidani, Luca Lorenzini, Federico Bianco, Claudio Rivetti, Silvia Catinella, Gino Villetti, Maurizio Civelli, Barbara Pioselli, Carlo Dani, Fabrizio Salomone

**Affiliations:** 1 Chiesi Farmaceutici, R&D Department, Parma, Italy; 2 Department of Drug Delivery, Helmholtz Institute for Pharmaceutical Research Saarland, Saarbrücken, Germany; 3 Department of Life Sciences, University of Parma, Parma, Italy; 4 Department of Neurosciences, Psychology, Drug Research and Child Health, Careggi University Hospital of Florence, Florence, Italy; University of Giessen Lung Center, GERMANY

## Abstract

Nasal continuous positive airway pressure (nCPAP) is a widely accepted technique of non-invasive respiratory support in spontaneously-breathing premature infants with respiratory distress syndrome (RDS). Surfactant administration techniques compatible with nCPAP ventilation strategy are actively investigated. Our aim is to set up and validate a respiratory distress animal model that can be managed on nCPAP suitable for surfactant administration techniques studies. Surfactant depletion was induced by bronchoalveolar lavages (BALs) on 18 adult rabbits. Full depletion was assessed by surfactant component analysis on the BALs samples. Animals were randomized into two groups: Control group (nCPAP only) and InSurE group, consisting of a bolus of surfactant (Poractant alfa, 200 mg/kg) followed by nCPAP. Arterial blood gases were monitored until animal sacrifice, 3 hours post treatment. Lung mechanics were evaluated just before and after BALs, at the time of treatment, and at the end of the procedure. Surfactant phospholipids and protein analysis as well as surface tension measurements on sequential BALs confirmed the efficacy of the surfactant depletion procedure. The InSurE group showed a significant improvement of blood oxygenation and lung mechanics. On the contrary, no signs of recovery were appreciated in animals treated with just nCPAP. The surfactant-depleted adult rabbit RDS model proved to be a valuable and efficient preclinical tool for mimicking the clinical scenario of preterm infants affected by mild/moderate RDS who spontaneously breathe and do not require mechanical ventilation. This population is of particular interest as potential target for the non-invasive administration of surfactant.

## Introduction

Pulmonary surfactant is a complex mixture of lipids and proteins that forms a thin layer that coats the inner surface of the alveoli. The main function of the surfactant system is to modulate the alveolar surface tension at the air-liquid interface avoiding the alveolar collapse at the end of the respiratory cycle[1]. In several pulmonary conditions such as the Respiratory Distress Syndrome (RDS) the surfactant system might be severely compromised leading to alveolar collapse, gas-exchange failure, inflammation, and eventually, to death[2]. Preterm infants are at high risk of developing neonatal RDS (nRDS), a condition characterized by a primary surfactant deficiency due to an incomplete lung maturation[3]. Surfactant replacement therapy (SRT), consisting on delivering a bolus of exogenous surfactant through the endotracheal tube of intubated infants[4], has traditionally been the first line treatment for nRDS[5, 6]. However, due to extended use of maternal steroids, which induce fetal lung maturation when administered before an imminent preterm birth, and to the inherent risks that intubation and mechanical ventilation through an endotracheal tube can cause in the fragile preterm lung, the use of non-invasive respiratory support, and in particular the administration of Continuous Positive Airway Pressure (CPAP) as a primary treatment option for nRDS, is gaining momentum[7, 8]. While the benefits of SRT and CPAP management are out of question, both therapies show a number of limitations that could be partly solved if both therapies could be administered simultaneously. Indeed, a concern in the universal application of CPAP to preterm infants is that those with moderate to severe nRDS will be inadequately supported by CPAP alone, ultimately will require intubation, and receive exogenous surfactant at a later than ideal time[9]. It has been reported that CPAP failure is primarily caused by untreated surfactant deficiency, and is associated with adverse outcomes, including increased risk of mortality as well as morbidities[10].

In the less invasive surfactant era, novel surfactant administration methods have been developed for coupling SRT with CPAP ventilation management. The InSurE technique (Intubate-Surfactant-Extubate) consists on intubating the babies just for surfactant administration, resuming to CPAP as soon as possible in order to reduce the exposure time of the babies to mechanical ventilation[11]. Although this approach is advocated and widely diffused for preterm infants with nRDS, intubation is an invasive procedure with attendant risks including oesophageal perforation, right main bronchus intubation, and clinical instability associated with accidental extubation episodes. Moreover, administration of intratracheal surfactant bolus has been associated with acute airway obstruction leading to apnea, transient hypoxia, bradycardia, hypotension, and reduced cerebral blood flow[12]. The full avoidance of intubation and mechanical ventilation by administering surfactant while the patients are managed in CPAP, either through a thin feeding tube[13, 14], or as an aerosol[15, 16], holds the potential to couple SRT and CPAP.

*In vivo* models have played a significant role in the preclinical development of SRT. On one hand, the depletion of the intrapulmonary surfactant pools by repeated bronco-alveolar lavages (BALs) with saline solution has been frequently used in juvenile as well as in adult rats[17, 18], rabbits[19, 20], and pigs[21, 22], among others, to implement successful animal models of acute pulmonary failure in the context of RDS. On the other hand, the intubated premature lamb has been the gold standard animal model to study the pathogenesis and the treatments of nRDS for many decades. This model adequately resembles the sequence of events that occur in human nRDS and has contributed to the discovery of SRT, its refinement, and to the development of new surfactant preparations and alternative delivery methods[23–27]. Both BAL-based models and the preterm lamb show a convenient mid-term survival, provided that the animals are intubated and extensive respiratory support is applied, especially in the case of untreated controls. Nevertheless, in the era of non-invasive surfactant, in order to be clinically relevant, the ventilation strategy of *in vivo* models must switch from the traditional use of invasive ventilation through a tracheal tube, towards the use of non-invasive support such as CPAP. In this regard, although feasible to some extent, managing preterm lambs in CPAP is complicated and the pulmonary outcomes after surfactant treatment are difficult to interpret due to the high variability in the response, and to the low sample size[28, 29]. In the case of BAL-based lung injury models the transition from invasive ventilation after the BAL procedure to non-invasive ventilation is certainly challenging and will depend, to a large extent, on the number of BALs performed to induce the lung injury[19, 20]. Our aim was to establish a BAL-based rabbit model spontaneously-breathing in nasal CPAP (nCPAP) with a severe respiratory distress that will further allow testing different strategies of non-invasive surfactant administration. For this purpose, respiratory distress was induced to 6–8 week old adult rabbits by repeated BALs and the animals were further managed in nCPAP for 3h through customized nasal prongs. To validate the model, we compared the pathophysiological outcomes of animals managed just with nCPAP to a group of animals treated with the InSurE approach. Additionally, we collected for representative animals sequential BALs performed during the lung lavage process. Their chemical composition was monitored by means of Liquid Chromatography-Mass Spectrometry (LC-MS) performing a qualitative and semi-quantitative analysis of surfactant protein (SP-B and SP-C) and main phospholipids. Moreover, the biophysical properties of the BALs were assessed with the Langmuir Blodgett (LB) approach. Both analyses confirmed the efficacy of the surfactant depletion procedure of our RDS model.

## Materials and Methods

### Animal handling and surfactant delivery protocol

The experiments were carried out in 6- to 7-week-old rabbits. The experimental procedure was approved by the intramural Animal Welfare Body and the Italian Ministry of Health (Prot.n°1300-2015-PR) and complied with the European and Italian regulations for animal care.

Rabbits (body weight of 1.5–2.5 kg) were initially sedated with intramuscular (i.m.) medetomidine (Domitor^®^, 2 mg/kg). The throat of the animals was first shaved and local anesthesia was applied in the anterior neck with lidocaine gel (Luan^®^ 2.5%). Thirty minutes later the animals received 50 mg/kg of ketamine (Imalgene^®^) and 5 mg/kg of xylazine (Rompun^®^) i.m. Rabbits, in supine position, were intubated and stabilized on positive pressure ventilation (Fabian HFO, Acutronic, Zug, Switzerland) with the following settings: FiO_2_ = 100%, Flow = 10 L/min, respiratory rate (RR) = 40 breaths/min, positive end-expiratory pressure (PEEP) = 3 cmH_2_O, tidal volume (V_T_) targeted to 7 ml/kg (with the peak inspiratory pressure, PIP, not exceeding 15 cmH_2_O) and inspiratory time of 0.5 sec. Airway flow, mean airway pressure (MAP) and V_T_ were monitored continuously with a flow sensor connected to the endotracheal tube. Body temperature was continuously monitored with a rectal probe and it was maintained by placing a heating pad underneath the animal. The pulse-oxymeter (RD SET Neo, Masimo, Irvine, CA) was attached to the leg of the animals in order to monitor oxygen saturation and heart rate.

After endotracheal intubation, a catheter was inserted into the right jugular vein for continuous infusion (100 μl/min) of 1 mg/ml of ketamine and 0.1 mg/ml of xylazine, while a second catheter was inserted into the right carotid artery for blood sampling. After instrumentation, blood gases were measured. If the initial inclusion criteria of arterial oxygen partial pressure (PaO_2_) value > 450 mmHg at PIP < 15 cmH_2_O were met, the animal was featured in the study and would undergo repeated BALs. BALs were performed by flushing the airways with 30 ml of pre-warmed (37°C) 0.9% NaCl solution, followed by a short recovery period in-between, until a PaO_2_ value < 150 mmHg was reached. Then, if after 15 min of stabilization in mechanical ventilation the respiratory failure was confirmed (PaO_2_ < 150mmHg, with PIP not exceeding 23 cmH_2_O) with an additional arterial blood gas analysis, the animal was extubated and managed in nCPAP, using customized nasal prongs (Fisher & Paykel, Auckland, New Zealand).

Once spontaneous breathing was established at a nCPAP level of 5 cmH_2_O, the animals were allocated to one of the two study groups: in the *nCPAP group* (n = 6) rabbits with surfactant deficiency induced by repeated BALs were maintained in nCPAP for 180 min. The animals allocated to the *InSurE group* (n = 9) received 200 mg/kg of Curosurf^®^ (Chiesi Farmaceutici, Parma, Italy) using the InSurE technique.[11] Briefly, a surfactant bolus was administered through the tracheal tube in about one minute and then the animals were immediately weaned to nCPAP and followed for 180 min. At the end of the experiment, animals were euthanized with an overdose of Penthotal^®^ 60mg/kg and a pressure/volume (P/V) curve was performed.

### Gas exchange and ventilation indices

**Fig 1:**
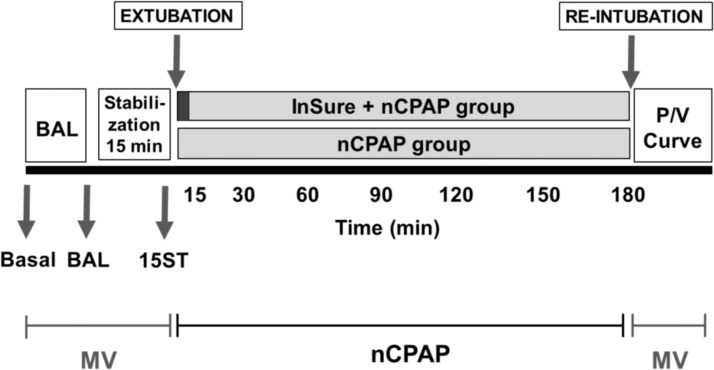
Scheme of the experimental set up. Scheme of the steps followed during the experimental procedure. Animals were intubated at baseline (Basal) and underwent several broncho-alveolar lavages (BAL) in order to induce surfactant depletion. A 15 min stabilization period was established to confirm the respiratory distress (15ST). In this period, animals were managed on mechanical ventilation (MV). After the respiratory distress was confirmed, animals were randomized to one of the experimental groups. Rabbits in the nCPAP group were maintained on nCPAP for 180 min. Rabbits in the InSurE group received 200 mg/kg of Curosurf^®^ and were then managed on nCPAP for 180 min. At the end of the experiment all rabbits were re-intubated to determine the pulmonary status. Post mortem a pressure/volume curve (P/V) was performed.

Arterial pH and blood gases were measured right after the induction of the anaesthesia, upon intubation (baseline), after inducing the respiratory distress by repeated BALs, and after the stabilization period following the insult to confirm the respiratory failure (15ST, Fig 1). Arterial blood gas analysis were also performed right after placing the animals on nCPAP, 15 and 30 min after the start of nCPAP, and then, every 30 min until the end of the experiment.

Alveolar-arterial oxygen pressure difference (A-aDO_2_) and arterial/alveolar ratio (a/ADO_2_) were calculated as:

$$A-aDO_{2}=P_{A}O_{2}-PaO_{2}$$ (1)

$$a/ADO_{2}=PaO_{2}/PiO_{2}-\left(PaCO_{2}/RQ\right)$$ (2)

where P_A_O_2_ is the alveolar oxygen concentration in mmHg, PiO_2_ is the partial pressure of inspired oxygen, PaCO_2_ is the arterial carbon dioxide partial pressure in mmHg, and R, the respiratory exchange quotient.

To allow a direct comparison of the pulmonary status at the end of the observational period, animals were re-intubated and managed on mechanical ventilation for a brief period of time, with exactly the same settings used at baseline (before the BALs: FiO_2_ 100%, Flow = 10 L/min; RR = 40 bpm, PEEP = 3 cmH_2_O, V_T_ targeted to 7 ml/kg with the initial PIP not exceeding 15 cmH_2_O, and inspiratory time of 0.5 sec). The ventilation efficiency index (VEI) was calculated to evaluate the overall ventilation efficiency of mechanically ventilated animals independently from the ventilation settings.

$$VEI=3800/\left[\left(PIP-PEEP\right)*RR*PaCO_{2}\right]$$ (3)

The oxygenation index (OI) was calculated to describe the severity of pulmonary dysfunction in ventilated animals.

$$OI=FiO_{2}*MAP*100/PaO_{2}$$ (4)

where MAP is the Mean Airway Pressure in cmH_2_O.

### Lung mechanics

The RR was determined by accurately counting the thorax movements of each animal for 10 sec. By multiplying this number by six, we expressed RR as number of breaths per minute. The RR was determined right after placing the animals on nCPAP, 15 and 30 min after nCPAP, and then, every 30 min until the end of the experiment. Values of dynamic compliance (C_dyn_) were measured at baseline, after BAL-induced respiratory distress, and again for comparison 180 min later, upon re-intubation, at the end of experiment. A P/V curve was also performed *post mortem* by progressively applying 5, 10, 15, 20, 25 and 30 ml of air-volume through a syringe and recording the pressure of the system at each volume point[30].

### Statistical analysis

All physiological data are presented as mean ± SEM. Raw data were analyzed and compared by repeated measures two-way analysis of variance (ANOVA) as a function of group and time, followed by Tukey’s t post-hoc test. Statistical analysis was performed using GraphPad software, version 6.0.

### LC-MS analysis of the BALs

BALs were collected by instillation of a pre-heated saline solution into the lungs and stored in 15 ml aliquots at -80°C. All the chemicals were MS grade and purchased from Sigma-Aldrich (unless otherwise stated).

Phospholipids were extracted with a mixture of chloroform/methanol (2:1). A volume of 500 μl of the BAL was added to 2 ml of extraction mixture and the solution was vortexed and centrifuged at 4000 rpm for 5 min at room temperature. The organic phase was collected, dried under nitrogen and re-suspended immediately before analysis with isopropanol/acetonitrile/water (2:1:1).

Surfactant proteins were extracted with a mixture of chloroform/methanol (3:2) added with 0.005 N trifluoroacetic acid. One ml aliquots of BAL were extracted twice with 2 ml of the extraction mixture. The samples were kept for 10 min at room temperature under mild agitation and then centrifuged (4000 rpm for 5 min). The organic phases were pooled together and dried under nitrogen. Before analysis they were re-suspended in 0.3% w/v sodium dodecyl sulphate. SP-C analysis was performed at this stage, while further processing was required for SP-B analysis and 10 μl were subjected to tryptic digestion. Proteolytic digestion was then performed using the In-Solution Tryptic Digestion and Guanidination Kit (Thermo Scientific) following the manufacturer instruction protocol. The samples were analysed by an UPLC Acquity binary system coupled to a high resolution Q-TOF (Synapt G2S, Waters). *Ad hoc* chromatographic separation and *ad hoc* mass spectrometric set up were used to determine the presence of phospholipids, SP-B and SP-C within the BALs.

#### Analytical method for phospholipids analysis

Phospholipids extracts were loaded onto a UPLC XSelect CSH130 C18 chromatographic column (2.5 μm, 4.6 x 150 mm). Phospholipids were eluted with a 20 minutes linear gradient from 50 to 99% mobile phase B at a flow rate of 0.4 ml/min (mobile phase A: 10 mM ammonium formate and 0.1% formic acid in acetonitrile 60/40 and mobile phase B: 10 mM ammonium formate and 0.1% formic acid in acetonitrile and isopropanol 10/90). Column temperature was set at 55°C. Both positive and negative polarities were used to ionize the samples according to which class of phospholipid was being determined. The spray voltage of the standard ESI source was set at 2 kV and 1 kV in positive and negative polarity, respectively. The source temperature was maintained at 100°C, while desolvation temperature was maintained at 250°C. The instrument was always operated in high resolution mode. LC-MS^e^ experiments were selected and both MS (low energy function) and MS/MS (high energy function; trap collision energy from 25 to 35) were collected within the m/z range 100–2000.

#### Analytical method for SP-B analysis

Tryptic digests were analysed by a Halo peptide-ES C18 UPLC chromatographic column (2.7 μm, 2.1 x 150 mm). Peptides were eluted from the column with a 100 minutes binary gradient at a flow rate of 0.2 ml/min from 1 to 90% mobile phase B (mobile phase A: water and 0.1% formic acid and mobile phase B: acetonitrile and 0.1% formic acid. Column temperature was set at 65°C. The spray voltage of the standard ESI source was set at 0.80 kV. The instrument was operated in positive polarity and high resolution mode. LC-MS^e^ experiments were selected and both MS (low energy function) and MS/MS (high energy function; trap collision energy from 25 to 35) were collected within the m/z range 50–2000.

#### Analytical method for SP-C analysis

The organic extracts supposed to contain intact SP-C was loaded onto a X-bridge BEH300 C4 chromatographic column (3.5 μm, 4.6 x 150 mm) with a 24 minutes binary gradient at a flow rate of 0.2 ml/min from 50 to 100% mobile phase B (mobile phase A: acetonitrile, water, and methanol 60/30/10 and 0.15% trifluoroacetic acid and mobile phase B: methanol and 0.15% trifluoroacetic acid. Column temperature was set at 65°C. The spray voltage of the standard ESI source was set at 3 kV. The instrument was operated in positive polarity and high resolution mode. LC-MS^e^ experiments were selected and both MS (low energy function) and MS/MS (high energy function; trap collision energy from 25 to 35) were collected within the m/z range 100–2000.

#### MS semi-quantitative data analysis

Data were acquired and elaborated by MassLynx 4.1 software and Biopharmalynx 1.3. Semi-quantitative data were accessed by manual peaks integration of the analytes of interest. Peaks corresponding to m/z 734.5, 721.5 and 747.5 corresponding to phosphatidyl-choline (PC; C16:0/C16:0), phosphatidyl-glycerol (PG; C16:0/C16:0) and PG (C16:0/C18:0) were extracted from the whole total ion chromatograms. Peaks representing intact SP-C protein were obtained by extraction of the [M+3H]^3+^charge state of SP-C at monoisotopic m/z 1386.1222 and its relative Met-Ox form at monoisotopic m/z 1391.4562. Finally, SP-B was identified and quantified by detection of the proteotypic peptide IQAMIPK peptide at m/z 421,77 [M+2H]^2+^ obtained by trypsin digestion of the whole intact protein. Typical chromatograms and high resolution spectra for all the investigated molecular species are available as supplementary information (Fig A to Fig E in S1 File).

### Langmuir Blodgett measurements

Freshly collected BALs were centrifuged at 250 x *g* for 10 min at 4°C to remove cells and debris. The centrifugation step was omitted in the case of Curosurf. A volume of 500 μl of the supernatant fraction was subjected to lipid extraction according to the Bligh and Dyer method[31]. The chloroform phase, containing the surfactant component, was used for Langmuir analysis.

Before each measurement, the ultra-pure (Milli-Q) water (≥18.2 MΩ-cm) subphase was quickly compressed to verify the absence of possible organic contaminations. The surfactant was spread onto the subphase of a Ribbon Barrier Trough (KSV NIMA, Finland) by means of a 100 μl glass microsyringe (Hamilton Company). The subphase operational area was 156 cm^2^ and the temperature was maintained constant at 37°C ± 1°C using an external circulating water bath.

Depending on the BALs phospholipid concentration, different volumes of the chloroform phase (ranging from 230 μl to 650 μl) were spread onto the subphase to reach a surface pressure of ≈ 15 mN/m. After spreading, the film was left undisturbed for 15 min to allow solvent evaporation. The film was then symmetrically compressed at a rate of 40 cm^2^/min. Surface pressure/area (π/A) isotherm was recorded in real time with the built-in software. For each sample, three or more independent measurements were performed.

#### Compressibility analysis of surfactant films

The two-dimensional compressibility of the BAL surfactant films was obtained from the compression isotherms. Film Compressibility (Cm) is defined as the inverse of the compression modulus and is given by:

Cm=−1/A*(δA/δπ) (5)

where A is the surface area and π the surface pressure. Cm provides information concerning phase transitions and fluidity/elasticity of the monolayer. Large Cm values are indicative of a state in which the film displays high compressibility and fluidity, whereas small Cm values reflect a high packing of the phospholipid molecules. For each measurement, Cm was plotted as function of the surface pressure π.

## Results

In the experimental procedure 6- to 7-week-old rabbits were randomly allocated either to the nCPAP-only group or to the InSurE-treatment group (Fig 1). Neither the body weight of the animals (nCPAP group: 1.72 ± 0.09 kg Vs. InSurE group: 1.7 ± 0.07 kg) nor the pH, PaO_2_, PaCO_2_, or C_dyn_ at baseline did significantly differ between groups. Respiratory failure was successfully induced by repeated BALs using 30 ml/kg of pre-warmed saline (37°C). Multiple lavages were performed for both groups: on average 7.3 ± 1.0 BALs were needed in the nCPAP group, whereas 5.1 ± 0.7 BALs were required in the InSurE group. The average number of lavages required in order to reach the respiratory failure criteria did not statistically differ among groups.

### Gas exchange and ventilation indices

**Fig 2:**
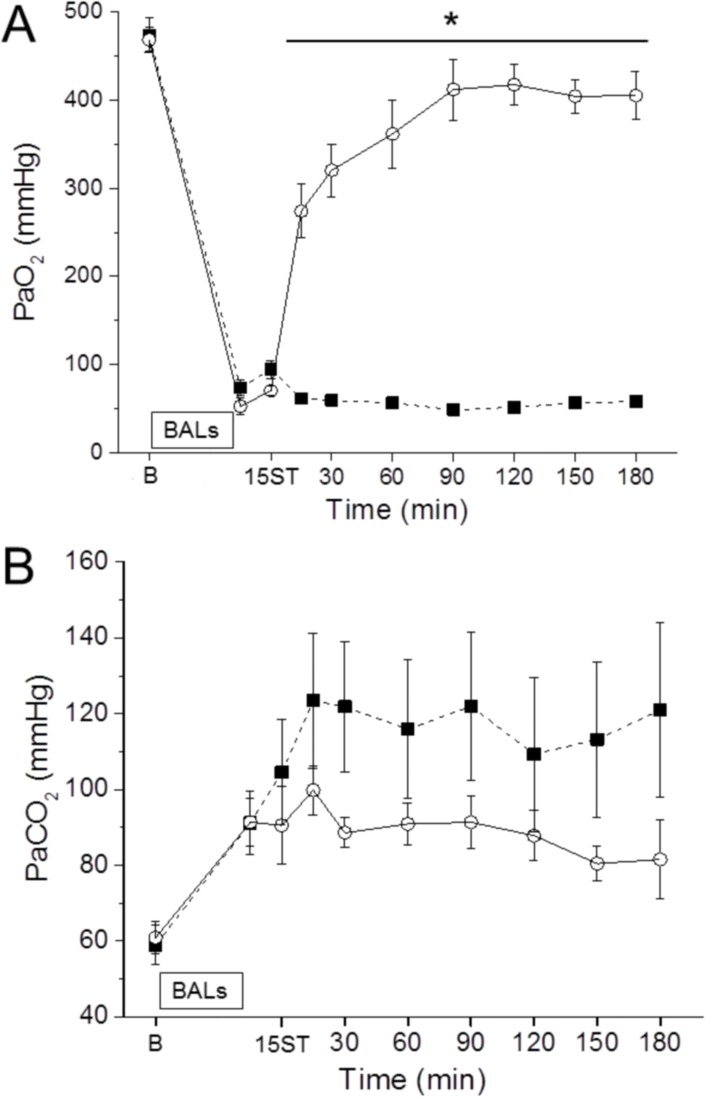
Gas exchange of surfactant-depleted rabbits. (A) Mean PaO_2_ and (B) PaCO_2_ values in animals managed just with nasal CPAP for 180 min (solid squares) and in animals treated with 200 mg/kg of surfactant following the InsSurE approach and managed further for 180 min in nasal CPAP (empty circles). There were no differences between the experimental groups before the induction of respiratory distress (baseline, B in the X-axis). The time point 15ST refers to the 15 min stabilization period established to confirm the respiratory distress induced by repeated broncho-alveolar lavages (BALs). N = 6 and N = 9 in the Control and InSurE groups, respectively. * P vs. Control < 0.05.

Blood gas samples were withdrawn at different time intervals in order to assess the pulmonary status. All animals had similar gas exchange and ventilation indices at baseline. However, after the induction of the respiratory failure (15ST) an abrupt drop of the arterial oxygenation and an increase of the PaCO_2_ were registered (Fig 2). Surfactant treatment was followed by a rapid and significant increase of PaO_2_ and was associated to lower levels of PaCO_2_ compared to the nCPAP group. The PaO_2_ curve in the nCPAP group remained a flat line with values below 100 mmHg at a FiO_2_ of 100%.

The ventilation indices were calculated taken into account additional ventilation parameters in addition to the arterial partial pressures of oxygen and carbon dioxide. The VEI and a/ADO_2_ decreased dramatically after the BALs (15ST), which was mirrored by a sudden increase of the OI and the A-aDO_2_ (Table 1). At the end of the observational period, all indices had significantly improved in the InSurE group compared to the nCPAP group.

**Table 1:** Ventilation indices and dynamic compliance of surfactant-depleted rabbits.

	GROUP	BASAL	RESPIRATORY DISTRESS	180 MIN
**A–aDO2**	Control	165 ± 21	488 ± 17^#^	504 ± 35
	InSurE	168 ± 11	529 ± 14^#^	244 ± 60^§^*
**A / aDO2**	Control	0.74 ± 0.03	0.16 ± 0.02^#^	0.10 ± 0.11
	InSurE	0.74 ± 0.02	0.15 ± 0.01^#^	0.68 ± 0.06^§^*
**VEI**	Control	0.22 ± 0.02	0.05 ± 0.01^#^	0.06 ± 0.02
	InSurE	0.23 ± 0.01	0.06 ± 0.01^#^	0.13 ± 0.02^§^*
**OI**	Control	1.2 ± 0.1	10.1 ± 1^#^	14.78 ± 2.23
	InSurE	1.1 ± 0	13.3 ± 1.1^#^	1.66 ± 0.14^§^*
**C**_**dyn**_ **(ml/cmH**_**2**_**O/kg)**	Control	0.88 ± 0.05	0.2 ± 0.02^#^	0.45 ± 0.09
	InSurE	1.09 ± 0.02	0.31 ± 0.04^#^	0.80 ± 0.04^§^*

Mean Alveolar-arterial Oxygen Pressure Difference (A-aDO2), Arterial/alveolar Ratio (a/ADO2), Ventilation Efficiency Index (VEI), Oxygenation Index (OI), and Dynamic Compliance (C_dyn_) in animals managed just with nasal CPAP (Control) and in animals treated with surfactant (InSurE) followed by nasal CPAP management. Values were recorded upon intubation (Basal), after the bronchoalveolar-lavage induced respiratory distress, and at the end of the experimental period (180 min). N = 6 and N = 9 in the Control and InSurE groups, respectively.

^#^ *P* vs. Basal values < 0.05;

^§^ *P* vs. Respiratory Distress Values < 0.05;

* *P* vs. Control Group < 0.05.

### Lung mechanics

C_dyn_ values were assessed at those time intervals in which the animals were ventilated with positive pressure ventilation, at baseline, after induction of the respiratory distress, and at the end of the observational period. C_dyn_ was equivalent for both groups at baseline. However, both parameters dropped dramatically after the induction of the respiratory distress and remained very low during the stabilization period. At the end of the experimental period, C_dyn_ had improved in both groups compared to the post-distress values. Nevertheless, C_dyn_ was significantly higher in the group of animals treated with the InSurE procedure in comparison to those animals treated with nCPAP only (Table 1).

**Fig 3:**
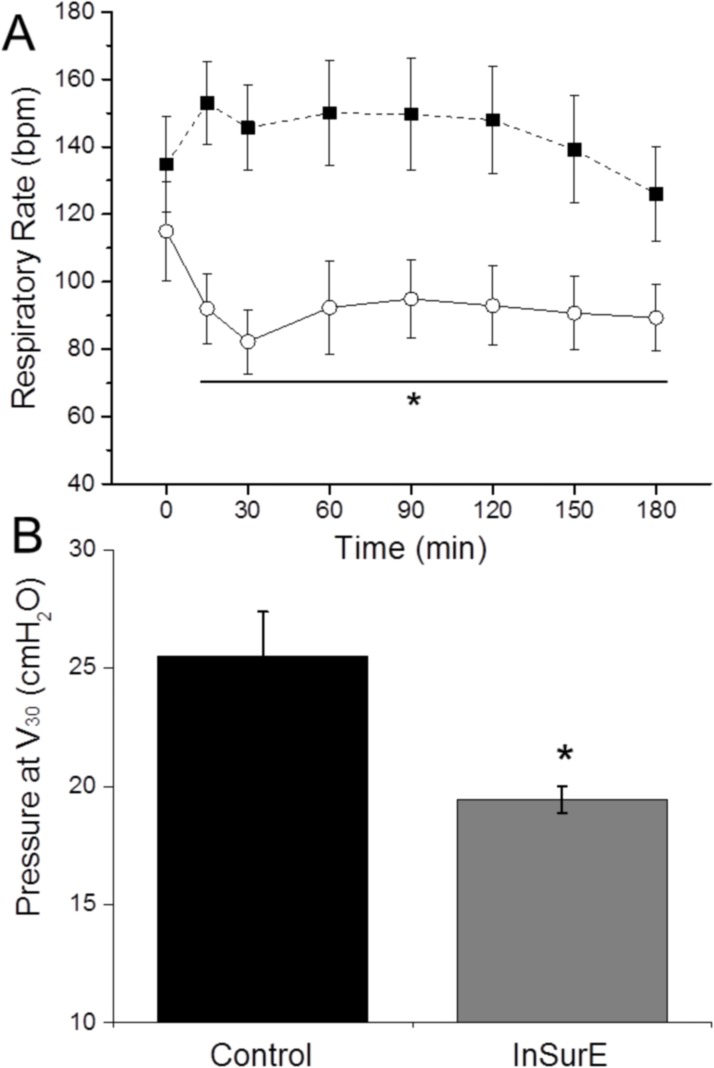
Respiratory parameters of surfactant-depleted rabbits. (A) Mean respiratory rate values of the animals managed just with nasal CPAP for 180 min (solid squares) and of the animals treated with 200 mg/kg of surfactant following the InsSurE approach and managed further for 180 min in nasal CPAP (empty circles). (B) Mean pressure values recorded after insufflating 30 ml of air *post mortem* (V30) in the lungs of the animals managed just with nasal CPAP (control) and of the animals treated with 200 mg/kg of surfactant following the InSurE approach and managed further for 180 min in nasal CPAP are shown.). N = 6 and N = 9 in the Control and InSurE groups, respectively. * P vs. Control < 0.05.

The RR was determined at regular intervals as soon as the animals were weaned from mechanical ventilation to nCPAP. RR values decreased within the first 30 min following the InSurE procedure, while the RR remained high and significantly different in the nCPAP group throughout the whole observational period (Fig 3A). A P/V curve was performed *post mortem*. The P/V curve was shifted to the right in the animals of nCPAP group, indicating a lower lung pulmonary compliance in this group (Fig F in S1 File). The mean pressure recorded after the insufflation of 30 ml of air to the lungs was significantly higher in the nCPAP group compared to the InSurE group, indicating a lower lung pulmonary compliance in the nCPAP group (Fig 3B).

### LC-MS analysis of the BALs

**Fig 4:**
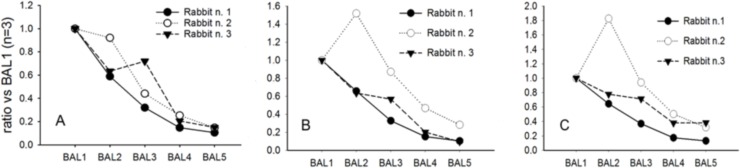
LC-MS analysis of phospholipids in bronchoalveolar lavages. LC-MS analysis of the **t**rends of phospholipid depletion upon sequential BALs. The results from three independent animals are shown. (A) PC (C16:0/C16:0), (B) PG (C16:0/C16:0) and (C) PG (C16:0/C18:0).

It is widely reported that relative changes in total ion intensities of analytes correlate well with their concentration changes in one sample *versus* another providing that they are analysed within similar experimental conditions. Three phospholipid molecules belonging to phosphatydilcholines and phosphatidylglycerols, the most abundant phospholipid classes within lung surfactant were monitored together with the two most relevant hydrophobic surfactant proteins, SP-B and SP-C. By the LC-MS methods described above, an exploratory semiquantitative determination of the surfactant-relevant lipids PC (C16:0/C16:0), PG (C16:0/C16:0) and PG (C16:0/C18:0), and the surfactant proteins SP-B and SP-C was successfully accomplished in between the sequential BALs of some exemplary animals. The relative amount of phospholipids showed a downward trend from the first to the fifth BALs performed during the respiratory distress induction process (Fig 4). The analysis of the fifth BAL (BAL5) revealed a dramatically lower phospholipid content relative to the first BAL (BAL1) in all animals, indicative of severe surfactant depletion.

**Fig 5:**
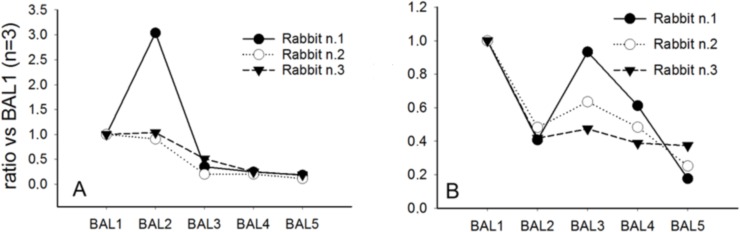
LC-MS analysis of surfactant proteins in bronchoalveolar lavages. LC-MS analysis of the **t**rends of surfactant protein depletion upon sequential BALs. The results from three independent animals are shown. (A) surfactant protein C and (B) surfactant protein B.

The depletion of surfactant proteins was also evaluated. The trends with respect to the BAL1 are reported for SP-C and SP-B (Fig 5). SP-C is considered a proteolipid molecule and the process of its depletion correlates well with the trend observed for phospholipid, reaching a significant depletion as observed in the BAL5. With regard to SP-B the pattern shows a linear decrease from BAL3 to BAL5 to a mean final reduction of SP-B of about 75% relative to BAL1. In our experimental conditions, after BAL5 a depletion of approximately 80% of the initial surfactant depot can be achieved, while more inter-animal variability is observed in the initial (BAL1 to BAL3) lavages.

### Langmuir Blodgett measurements

**Fig 6:**
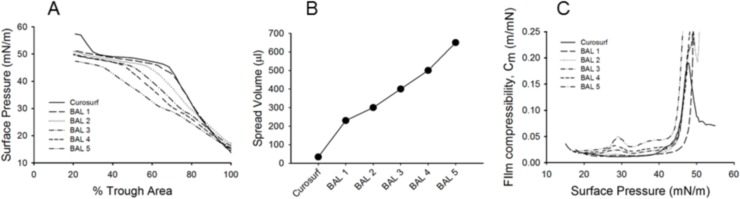
Langmuir Blodgett analysis of bronchoalveolar lavages. (A) Surface pressure versus % Trough area compression isotherms of Curosurf^®^ and the sequential broncho-alveolar lavages (BALs). The surfactant films were spread on pure water subphase at 37°C ± 1°C and compressed at a rate of 40 cm^2^/min. Each curve gives the data from a single experiment representative of three replicates. (B) Volume of Curosurf and BALs spread at the air-water interface to reach a surface pressure of ≈ 15 mN/m. C) Film Compressibility (Cm) of Curosurf and BALs as a function of surface pressure, *π*. Cm was determined from compression isotherm using the equation: Cm = -1/A*(dA/d*π*).

LB technique was employed to analyze the evolution of surfactant depletion process upon sequential BALs. It is widely known that the biophysical properties of surfactant depend on the chemical composition. In the range 15–45 mN/m of the Curosurf isotherm, phospholipid chains with a low (liquid-expanded phase) and high packing (liquid-condensed phase) coexist throughout the compression, up to the plateau, which represents the monolayer to multi-layer transition and the exclusion of the more fluid-like and unsaturated lipids (squeeze-out). Further compression to surface pressure > 45 mN/m promotes the monolayer enrichment in saturated phospholipids, which become organized in very condensed phases[32]. Despite the shape of the BAL isotherms was partially similar to that of Curosurf isotherm, for BAL 1 in particular, some differences in both chemical composition and behavior at the air-water-interface of the sequential BALs can be deduced. On the one hand, a progressive reduction of the isotherm slope in the pressure range 15–45 mN/m was detected from BAL 2 to BAL 5, with respect to Curosurf and BAL 1 (Fig 6A). Considering that the isotherm slope in this pressure range is representative of the packing density of the phospholipid chains into the monolayer[33, 34], it seems that sequential BALs become gradually organized in more fluid films. On the other hand, the required spread volume of the samples to reach a surface pressure of 15 mN/m varied significantly (Fig 6B). Indeed, an increase in the spread volume of about 3-fold between BAL 1 and BAL 5 and of about 20-fold between Curosurf and BAL 5 was recorded. In agreement with the results obtained with the LC-MS approach, these data might suggest that the phospholipid components were progressively reduced form BAL 1 to BAL 5, indicating surfactant depletion in animals, as a consequence of the BALs.

Finally, film compressibility (Cm), which is a measure of the resistance to compression of the surfactant monolayer, indicated the more fluid organization of the sequential BALs. Indeed, larger compressibility values were obtained for all BALs with respect to Curosurf, in particular at pressures > 45 mN/m (Fig 6C). In this pressure range, BAL films were not organized in a tightly-packed phospholipid phase and could not be further compressed after the plateau formation, at variance with Curosurf, as indicated by the compression isotherms.

Because more fluid monolayers are promoted by the abundance of unsaturated phospholipids and/or cholesterol[35, 36], an enrichment of these molecules could occur in the chemical composition of sequential BALs. Thus, the saturated phospholipid component might be primarily removed in the first lavages as determined by LC-MS analysis.

## Discussion

In the present work we set-up and validated an *in vivo* model of respiratory distress that can be managed in nCPAP with customized nasal prongs. It represents a useful tool to study non-invasive procedures for surfactant delivery in the contest of RDS. Furthermore, we also performed a LC-MS analysis and a LB analysis of the sequential BALs collected from representative animals during the respiratory distress induction process. Both analyses demonstrate that surfactant depletion is a progressive process involving a gradual removal of the main surfactant components, SP-C and SP-B proteins and phospholipids. The data collected over a significant number of animals suggested that at least 5 BALs are necessary to achieve a marked reduction of the main surfactant components. The BAL analysis correlated well with the low and steady oxygenation levels in the nCPAP group. In this group, the use of nCPAP alone could not reverse the respiratory failure. On the other hand, surfactant treatment with the InSurE technique significantly improved the overall pulmonary status and served as a proof of concept group to validate the model.

SRT set a milestone in the treatment of nRDS after its worldwide approval in the early 90s. However, due to the risks associated to the endotracheal intubation and mechanical ventilation[37], which are pre-requisites to deliver a surfactant bolus to the lungs, the use of SRT as a primary treatment for RDS is being challenged by non-invasive ventilation methods such as nCPAP[7, 8]. The benefits of nCPAP include the stabilization of the alveoli at the end of the expiratory phase, yet avoiding the tracheal intubation and its associated risks. However, CPAP failure is relatively frequent and increases with decreasing the gestational age of RDS infants[10]. SRT is more effective when administered early in the course of RDS and thus, administering SRT after the CPAP failure is detected may reduce the benefits of therapy[38]. A way to simultaneously apply both therapies has been pursued for a long time. For instance, aerosol delivery of surfactant has been shown to improve lung function in animal models and it has been attempted in combination with non-invasive ventilation in clinical studies[16, 39, 40]. The clinical results of these studies did not find a clear advantage of aerosolized surfactant so far and this delivery method remains under active preclinical development[28, 41, 42]. The InSurE technique was successfully introduced by Verder *et al*. and aimed at reducing the mechanical ventilation time of RDS infants[11]. More recently the intratracheal administration of surfactant avoiding the traditional tracheal intubation by advancing a thin tube through the vocal cords, while the infants are managed on CPAP has been shown to be a feasible alternative the classic SRT[13, 14, 43]. In the non-invasive surfactant era, novel animal models are required to address new questions regarding the safety and the efficacy of alternative surfactant administration methods. Most importantly, the animal model should be managed on non-invasive respiratory support such as CPAP in order to mimic the current trends in neonatal care[7].

Animal models have played a significant role in the development of SRT as well as in the efficacy-testing of different surfactant preparations. The intubated preterm lamb model has been considered the *gold standard* model to study nRDS[23, 25, 27, 29], because the primary surfactant deficiency in the lambs adequately resembles human nRDS. The induction of lung injury by repeated BALs has also been used extensively as a model of acute lung-injury in the contest of RDS[12, 17, 18, 22, 44]. Nevertheless, the majority of these studies were performed during tracheal intubation and mechanical ventilation with full control of the airway. Managing preterm lambs in CPAP has been shown to be feasible in lambs with mild to moderate RDS, although pulmonary outcomes after surfactant treatment in combination with non-invasive ventilation are difficult to interpret due to the high variability in the response to CPAP and surfactant, and to the low sample size of lamb studies[28, 29]. BAL-based *in vivo* models of respiratory distress have also been managed in CPAP with conflicting results. Dijk *et al*. treated lung-lavaged rabbits with either instilled or nebulized surfactant during mechanical ventilation, and used animals treated with nebulized saline as controls[12]. After two hours of mechanical ventilation and 30 min of synchronized intermittent mechanical ventilation, the protocol mandated to wean the animals to CPAP. The gas exchange rapidly deteriorated in the surfactant-treated groups and none of the saline-treated animals survived the weaning to CPAP. On the contrary, Walther *et al*. treated lung-lavaged rabbits managed on CPAP with surfactant aerosols and found a rapid and sustained recovery of the oxygenation with PaO_2_ values around 300 mmHg at a FiO_2_ of 100%[19]. Unfortunately, this study did not include a control group of animals treated with nCPAP only and it did not consider a lag time between the end of depletion procedure and the extubation/treatment procedure. The number of the BALs performed to induce the insult in the Dijk and Walther *et al*. studies was 5 and 3, respectively. In our study we performed at least 5 BALs per animal, which produced a severe respiratory distress as indicated by the poor gas exchange and the low C_dyn_ values observed in the nCPAP group. Consistently with the aforementioned studies, the biochemical analysis of the sequential BALs recovered from representative animals during the respiratory distress induction process revealed a high variability in the relative amount of the surfactant-relevant phospholipids and proteins among the different animals in BALs 2 and 3. However, after 5 BALs the amount of surfactant phospholipids and proteins analyzed here dropped dramatically and the inter-animal variability was reduced, suggesting successful surfactant depletion. Moreover, the biophysical analysis of the sequential BALs shows that the slope of the isotherm decreases in sequential BALs, suggesting that the chemical composition from BAL1 to BAL5 progressively changes from an intrapulmonary situation in which the saturated phospholipids dominate (BALs 1 and 2), to a new scenario where the unsaturated phospholipids and/or cholesterol predominate[35, 36]. This hypothesis correlates well with the LC-MS analysis, which reveals that the saturated phospholipid component is being primarily removed in the first BALs.

The detected high variability of surfactant-relevant components between BALs 2 and 3 suggests that in some animals surfactant depletion might not be completed with just 3 BALs and that, as a consequence, the lung function of some animals might partially improve by just applying nCPAP. From an experimental point of view, our data strongly suggest that it may be difficult to distinguish the contribution to the pulmonary function improvement of the nCPAP from that of the aerosolized surfactant if just 3 BALs are performed. Indeed, a partial recovery of the oxygenation in rabbits managed in nCPAP that just underwent 3 BALs, without a lag time application before extubation (Fig G in S1 File). In the model described here the animals did not recover from the insult (5 BALs) if they were merely managed with nCPAP, whereas in the InSurE group surfactant treatment was effective in improving arterial oxygen levels to near baseline values.

Ultimately, several limitations of the model must be acknowledged. 1) After the induction of the respiratory distress by repeated BALs the PaCO_2_ values were high and remained elevated during the experimental period, even in those animals treated with a fully effective surfactant dose. This exacerbated hypercarbia derives from the severe respiratory distress induced by the BALs, but can be also due to the use of human nasal cannulas to provide the nCPAP, which are not designed for the rabbits’ anatomy, and might have increased the airway resistance, accounting for a high PaCO_2_, even after surfactant treatment. Moreover, the respiratory drive of the animals might have been attenuated by the action of the anesthesia accounting also for high PaCO_2_ levels. Nevertheless, this issue can be improved in the present model with the administration of a pH buffer such as THAM or bicarbonate in the course of the depletion procedure. 2) Although the rabbit model has the potential to reduce the variability of the surfactant treatment response compared to a premature model, it also misses two important nRDS features related to the prematurity: lung architecture still in development and an immature molecular and biochemical metabolism.

In conclusion, we have set up and validated the lung-lavaged spontaneously-breathing rabbit on nCPAP as a model for RDS. The biophysical and biochemical analysis performed in the sequential BALs shows that the surfactant depletion protocol applied in the present study, in which at least 5 BALs were performed per animal, yielded a significant respiratory distress. The dramatic drop of the surfactant–relevant phospholipids and surfactant proteins, as shown by LC-MS, correlated well with the significantly lower lung compliance and the poor oxygenation observed in the nCPAP group, yet allowing this group of animals to be ventilated with non-invasive support. The broad difference in terms of oxygenation and lung mechanics observed between the nCPAP and the InSurE groups and the affinity with clinical nCPAP setting indicate that this animal model is a suitable tool for testing non-invasive surfactant delivery techniques where surfactant deposition is generally low. In this scenario, reliable models are required in which the exogenous surfactant effect must be clearly differentiated from a potential spontaneous recovery.

## Supporting Information

S1 File This file contains the following figures: Fig A. Typical extracted ion chromatogram (Panel A) and relative spectrum under the peak (Panel B) showing the signal attributed to the phospholipid phosphatidyl-choline (PC; C16:0/C16:0). Fig B. Typical extracted ion chromatogram (Panel A) and relative spectrum under the peak (Panel B) showing the signal attributed to the phospholipid phosphatidyl-glycerol (PG; C16:0/C16:0). Fig C. Typical extracted ion chromatogram (Panel A) and relative spectrum under the peak (Panel B) showing the signal attributed to the phospholipid phosphatidyl-glycerol PG (C16:0/C18:0). Fig D. Typical extracted ion chromatograms and relative spectrum under the peaks (Panel C) showing the signal attributed to the [M+3H]3+charge state of SP-C (monoisotopic m/z 1386.1222, Panel A) and to the [M+3H]3+charge state of its relative Met-Ox (monoisotopic m/z 1391.4562, Panel B). Fig E. Typical extracted ion chromatogram (Panel A) and relative spectrum under the peak (Panel B) showing the signal attributed to the [M+2H]^2+^ charge state of the proteotypic peptide IQAMIPK at m/z 421,77 obtained by trypsin digestion of the whole SpB intact protein. Fig F. Representative Pressure/Volume curves of surfactant-treated and untreated surfactant depleted rabbits. Pressure/Volume curves (P/V) performed *post mortem* in rabbits with respiratory failure induced by repeated broncho-alveolar lavages just managed with nCPAP (green curve), and in animals treated with a clinical dose of surfactant (Curosurf^®^, 200 mg/kg) delivered following the InSurE approach (surfactants instillation through the endotracheal tube followed by CPAP, red curve). The P/V curve was performed by progressively applying 5, 10, 15, 20, 25 and 30 ml of air-volume through a syringe and recording the pressure of the system at each volume point. Fig G. Mean PaO_2_ in rabbits managed in CPAP after bronco-alveolar lavages. Mean PaO_2_ values in rabbits managed with nasal CPAP for 180 min that underwent at least 5 broncho-alveolar lavages (BALs, black squares), in rabbits managed with nasal CPAP for 180 min that just underwent 3 BALs (blue triangles) and in rabbits that underwent at least 5 BALs (red circles) treated with 200 mg/kg of surfactant (Curosurf^®^) following the InsSurE approach (surfactants instillation through the endotracheal tube followed by CPAP) and further managed for 180 min in nasal CPAP. Arterial blood gases were measured right after the induction of the anesthesia, upon intubation (baseline, B), after inducing the respiratory distress by repeated BALs and placing the animals on CPAP (0 min), 15 and 30 min after the start of CPAP ventilation, and then, every 30 min until the end of the experiment. Mean ± SEM are shown.
(DOCX)

S1 Dataset This dataset contains the individual arterial oxygen and carbon dioxide partial pressure (PaO_2_ and PaCO_2_), pH, respiratory rate (RR), and dynamic compliance (Cdyn) values of each animal included in this study. (XLSX)

## References

[1] Perez-Gil J, Weaver TE. Pulmonary surfactant pathophysiology: current models and open questions. Physiology (Bethesda). 2010;25: 132–141. 20551227 10.1152/physiol.00006.2010

[2] Matthay MA, Zemans RL. The acute respiratory distress syndrome: pathogenesis and treatment. Annu Rev Pathol. 2011;6: 147–163. 10.1146/annurev-pathol-011110-130158 20936936 PMC3108259

[3] Avery ME, Mead J. Surface properties in relation to atelectasis and hyaline membrane disease. AMA J Dis Child. 1959;97: 517–523. 13649082 10.1001/archpedi.1959.02070010519001

[4] Schmolzer GM, Kamlin CO, Dawson JA, Morley CJ, Davis PG. Tidal volume delivery during surfactant administration in the delivery room. Intensive Care Med. 2011;37: 1833–1839. 10.1007/s00134-011-2366-2 21976187

[5] Walsh BK, Daigle B, DiBlasi RM, Restrepo RD, American Association for Respiratory C. AARC Clinical Practice Guideline. Surfactant replacement therapy: 2013. Respir Care. 2013;58: 367–375. 10.4187/respcare.02189 23359726

[6] Sweet DG, Carnielli V, Greisen G, Hallman M, Ozek E, Plavka R, et al European consensus guidelines on the management of neonatal respiratory distress syndrome in preterm infants—2010 update. Neonatology. 2010;97: 402–417. 10.1159/000297773 20551710

[7] Rojas-Reyes MX, Morley CJ, Soll R. Prophylactic versus selective use of surfactant in preventing morbidity and mortality in preterm infants. Cochrane Database Syst Rev. 2012: CD000510 10.1002/14651858.CD000510.pub2 22419276 PMC12034442

[8] Blennow M, Bohlin K. Surfactant and noninvasive ventilation. Neonatology. 2015;107: 330–336. 10.1159/000381122 26044100

[9] Dargaville PA, Gerber A, Johansson S, De Paoli AG, Kamlin CO, Orsini F, et al Incidence and Outcome of CPAP Failure in Preterm Infants. Pediatrics. 2016;138. 10.1542/peds.2015-3985 27365307

[10] Dargaville PA, Aiyappan A, De Paoli AG, Dalton RG, Kuschel CA, Kamlin CO, et al Continuous positive airway pressure failure in preterm infants: incidence, predictors and consequences. Neonatology. 2013;104: 8–14. 10.1159/000346460 23595061

[11] Verder H, Robertson B, Greisen G, Ebbesen F, Albertsen P, Lundstrom K, et al Surfactant therapy and nasal continuous positive airway pressure for newborns with respiratory distress syndrome. Danish-Swedish Multicenter Study Group. N Engl J Med. 1994;331: 1051–1055. 10.1056/NEJM199410203311603 8090164

[12] Dijk PH, Heikamp A, Bambang Oetomo S. Surfactant nebulisation prevents the adverse effects of surfactant therapy on blood pressure and cerebral blood flow in rabbits with severe respiratory failure. Intensive Care Med. 1997;23: 1077–1081. 9407244 10.1007/s001340050459

[13] Dargaville PA, Aiyappan A, Cornelius A, Williams C, De Paoli AG. Preliminary evaluation of a new technique of minimally invasive surfactant therapy. Arch Dis Child Fetal Neonatal Ed. 2011;96: F243–248. 10.1136/adc.2010.192518 20971722

[14] Gopel W, Kribs A, Ziegler A, Laux R, Hoehn T, Wieg C, et al Avoidance of mechanical ventilation by surfactant treatment of spontaneously breathing preterm infants (AMV): an open-label, randomised, controlled trial. Lancet. 2011;378: 1627–1634. 10.1016/S0140-6736(11)60986-0 21963186

[15] Mazela J, Merritt TA, Finer NN. Aerosolized surfactants. Curr Opin Pediatr. 2007;19: 155–162. 10.1097/MOP.0b013e32807fb013 17496758

[16] Jorch G, Hartl H, Roth B, Kribs A, Gortner L, Schaible T, et al Surfactant aerosol treatment of respiratory distress syndrome in spontaneously breathing premature infants. Pediatr Pulmonol. 1997;24: 222–224. 9330420 10.1002/(sici)1099-0496(199709)24:3<222::aid-ppul9>3.0.co;2-o

[17] Bahlmann H, Sun B, Nilsson G, Curstedt T, Robertson B. Aerosolized surfactant in lung-lavaged adult rats: factors influencing the therapeutic response. Acta Anaesthesiol Scand. 2000;44: 612–622. 10786751 10.1034/j.1399-6576.2000.00521.x

[18] Alvarez FJ, Alfonso LF, Gastiasoro E, Lopez-Heredia J, Arnaiz A, Valls-i-Soler A. The effects of multiple small doses of exogenous surfactant on experimental respiratory failure induced by lung lavage in rats. Acta Anaesthesiol Scand. 1995;39: 970–974. 8848901 10.1111/j.1399-6576.1995.tb04207.x

[19] Walther FJ, Hernandez-Juviel JM, Waring AJ. Aerosol delivery of synthetic lung surfactant. PeerJ. 2014;2: e403 10.7717/peerj.403 24918030 PMC4045332

[20] Dijk PH, Heikamp A, Bambang Oetomo S. Surfactant nebulisation: lung function, surfactant distribution and pulmonary blood flow distribution in lung lavaged rabbits. Intensive Care Med. 1997;23: 1070–1076. 9407243 10.1007/s001340050458

[21] Kandler MA, von der Hardt K, Schoof E, Dotsch J, Rascher W. Persistent improvement of gas exchange and lung mechanics by aerosolized perfluorocarbon. Am J Respir Crit Care Med. 2001;164: 31–35. 10.1164/ajrccm.164.1.2010049 11435235

[22] Roberts KD, Lampland AL, Meyers PA, Worwa CT, Plumm BJ, Mammel MC. Laryngeal mask airway for surfactant administration in a newborn animal model. Pediatr Res. 2010;68: 414–418. 10.1203/PDR.0b013e3181ef7619 20613684

[23] Adams FH, Towers B, Osher AB, Ikegami M, Fujiwara T, Nozaki M. Effects of tracheal instillation of natural surfactant in premature lambs. I. Clinical and autopsy findings. Pediatr Res. 1978;12: 841–848. 10.1203/00006450-197808000-00008 581094

[24] Jobe A, Jacobs H, Ikegami M, Jones S. Cardiovascular effects of surfactant suspensions given by tracheal instillation to premature lambs. Pediatr Res. 1983;17: 444–448. 10.1203/00006450-198306000-00002 6553821

[25] Gastiasoro-Cuesta E, Alvarez-Diaz FJ, Rey-Santano C, Arnaiz-Renedo A, Loureiro-Gonzalez B, Valls-i-Soler A. Acute and sustained effects of lucinactant versus poractant-alpha on pulmonary gas exchange and mechanics in premature lambs with respiratory distress syndrome. Pediatrics. 2006;117: 295–303. 10.1542/peds.2005-0378 16452346

[26] Rey-Santano C, Mielgo VE, Andres L, Ruiz-del-Yerro E, Valls-i-Soler A, Murgia X. Acute and sustained effects of aerosolized vs. bolus surfactant therapy in premature lambs with respiratory distress syndrome. Pediatr Res. 2013;73: 639–646. 10.1038/pr.2013.24 23403804

[27] Sato A, Ikegami M. SP-B and SP-C containing new synthetic surfactant for treatment of extremely immature lamb lung. PLoS One. 2012;7: e39392 10.1371/journal.pone.0039392 22808033 PMC3396642

[28] Hutten MC, Kuypers E, Ophelders DR, Nikiforou M, Jellema RK, Niemarkt HJ, et al Nebulization of Poractant alfa via a vibrating membrane nebulizer in spontaneously breathing preterm lambs with binasal continuous positive pressure ventilation. Pediatr Res. 2015;78: 664–669. 10.1038/pr.2015.165 26322413

[29] Rahmel DK, Pohlmann G, Iwatschenko P, Volland J, Liebisch S, Kock H, et al The non-intubated, spontaneously breathing, continuous positive airway pressure (CPAP) ventilated pre-term lamb: a unique animal model. Reprod Toxicol. 2012;34: 204–215. 10.1016/j.reprotox.2012.05.089 22659287

[30] Wada K, Jobe AH, Ikegami M. Tidal volume effects on surfactant treatment responses with the initiation of ventilation in preterm lambs. J Appl Physiol (1985). 1997;83: 1054–1061. 9338410 10.1152/jappl.1997.83.4.1054

[31] Bligh EG, Dyer WJ. A rapid method of total lipid extraction and purification. Can J Biochem Physiol. 1959;37: 911–917. 10.1139/o59-099 13671378

[32] Zhang H, Wang YE, Fan Q, Zuo YY. On the low surface tension of lung surfactant. Langmuir. 2011;27: 8351–8358. 10.1021/la201482n 21650180 PMC4849879

[33] Nowotarska SW, Nowotarski KJ, Friedman M, Situ C. Effect of structure on the interactions between five natural antimicrobial compounds and phospholipids of bacterial cell membrane on model monolayers. Molecules. 2014;19: 7497–7515. 10.3390/molecules19067497 24914896 PMC6271777

[34] Moore BGK, M C., Akamatsu S.; Rondelez F.. Phase Diagram of Langmuir Monolayers of Pentadecanoic Acid: Quantitative Comparison of Surface Pressure and Fluorescence Microscopy Results. J Phys Chem. 1990;94: 4588–4595.

[35] Bringezu F D J; Brezesinski G.; Zasadzinski J.A. Changes in Model Lung Surfactant Monolayers Induced by Palmitic Acid. Langmuir. 2001;17: 4641–4648.

[36] Zhang H, Wang YE, Neal CR, Zuo YY. Differential effects of cholesterol and budesonide on biophysical properties of clinical surfactant. Pediatr Res. 2012;71: 316–323. 10.1038/pr.2011.78 22391630 PMC3338335

[37] Hillman NH, Moss TJ, Kallapur SG, Bachurski C, Pillow JJ, Polglase GR, et al Brief, large tidal volume ventilation initiates lung injury and a systemic response in fetal sheep. Am J Respir Crit Care Med. 2007;176: 575–581. 10.1164/rccm.200701-051OC 17641159 PMC1994225

[38] Seidner SR, Ikegami M, Yamada T, Rider ED, Castro R, Jobe AH. Decreased surfactant dose-response after delayed administration to preterm rabbits. Am J Respir Crit Care Med. 1995;152: 113–120. 10.1164/ajrccm.152.1.7599809 7599809

[39] Berggren E, Liljedahl M, Winbladh B, Andreasson B, Curstedt T, Robertson B, et al Pilot study of nebulized surfactant therapy for neonatal respiratory distress syndrome. Acta Paediatr. 2000;89: 460–464. 10830460 10.1080/080352500750028195

[40] Finer NN, Merritt TA, Bernstein G, Job L, Mazela J, Segal R. An open label, pilot study of Aerosurf(R) combined with nCPAP to prevent RDS in preterm neonates. J Aerosol Med Pulm Drug Deliv. 2010;23: 303–309. 10.1089/jamp.2009.0758 20455772

[41] Goikoetxea E, Murgia X, Serna-Grande P, Valls-i-Soler A, Rey-Santano C, Rivas A, et al In vitro surfactant and perfluorocarbon aerosol deposition in a neonatal physical model of the upper conducting airways. PLoS One. 2014;9: e106835 10.1371/journal.pone.0106835 25211475 PMC4161382

[42] Lampland AL, Wolfson MR, Mazela J, Henderson C, Gregory TJ, Meyers P, et al Aerosolized KL4 surfactant improves short-term survival and gas exchange in spontaneously breathing newborn pigs with hydrochloric acid-induced acute lung injury. Pediatr Pulmonol. 2014;49: 482–489. 10.1002/ppul.22844 24039229

[43] Kanmaz HG, Erdeve O, Canpolat FE, Mutlu B, Dilmen U. Surfactant administration via thin catheter during spontaneous breathing: randomized controlled trial. Pediatrics. 2013;131: e502–509. 10.1542/peds.2012-0603 23359581

[44] Lachmann B, Robertson B, Vogel J. In vivo lung lavage as an experimental model of the respiratory distress syndrome. Acta Anaesthesiol Scand. 1980;24: 231–236. 7445941 10.1111/j.1399-6576.1980.tb01541.x

