# Supplementary material for: Physiological, Biochemical, and Biophysical Characterization of the Lung-Lavaged Spontaneously-Breathing Rabbit as a Model for Respiratory Distress Syndrome
Source: PLoS One. 2017 Jan 6;12(1):e0169190. doi: 10.1371/journal.pone.0169190 (PMC5217971; doi:10.1371/journal.pone.0169190)

**Physiological, biochemical, and biophysical characterization of the lung-lavaged spontaneously-breathing rabbit as a model for respiratory distress syndrome**

**Supporting Information**

Ricci F^1^, Catozzi C^1^, Murgia X^2^, Rosa B^1^, Amidani D^3^, Lorenzini L^1^, Bianco F^1^, Rivetti C^3^, Catinella S^1^, Villetti G^1^, Civelli M^1^, Pioselli B^1^, Dani C^4^, Salomone F^1^

^1^Chiesi Farmaceutici, R&D Department, Largo Belloli 11/A, IT-43122, Parma, Italy.

^2^Department of Drug Delivery, Helmholtz Institute for Pharmaceutical Research Saarland, Saarbrücken, Germany.

^3^Department of Life Sciences University of Parma Parco Area delle Scienze 23/A 43124 Parma Italy.

^4^Department of Neurosciences, Psychology, Drug Research and Child Health, Careggi University Hospital of Florence, Florence, Italy.

**Corresponding author:** Fabrizio Salomone, PhD. Largo Francesco Belloli, 11/A 43122,Parma, ITALY; Tel. +39 0521 1689158. E-mail. f.salomone@chiesi.com

**Fig A.** Typical extracted ion chromatogram (Panel A) and relative spectrum under the peak (Panel B) showing the signal attributed to the phospholipid phosphatidyl-choline (PC; C16:0/C16:0).

**
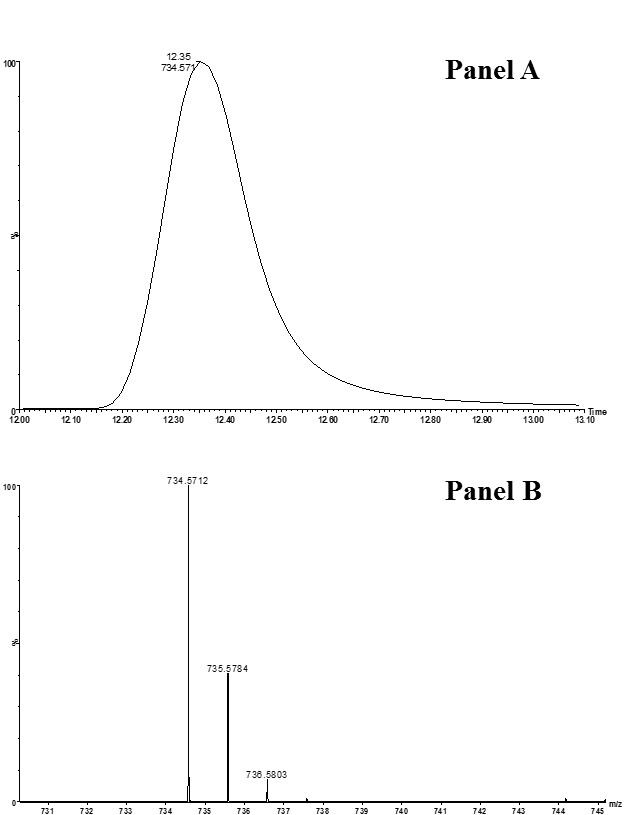
**

**Fig B.** Typical extracted ion chromatogram (Panel A) and relative spectrum under the peak (Panel B) showing the signal attributed to the phospholipid phosphatidyl-glycerol (PG; C16:0/C16:0).

**
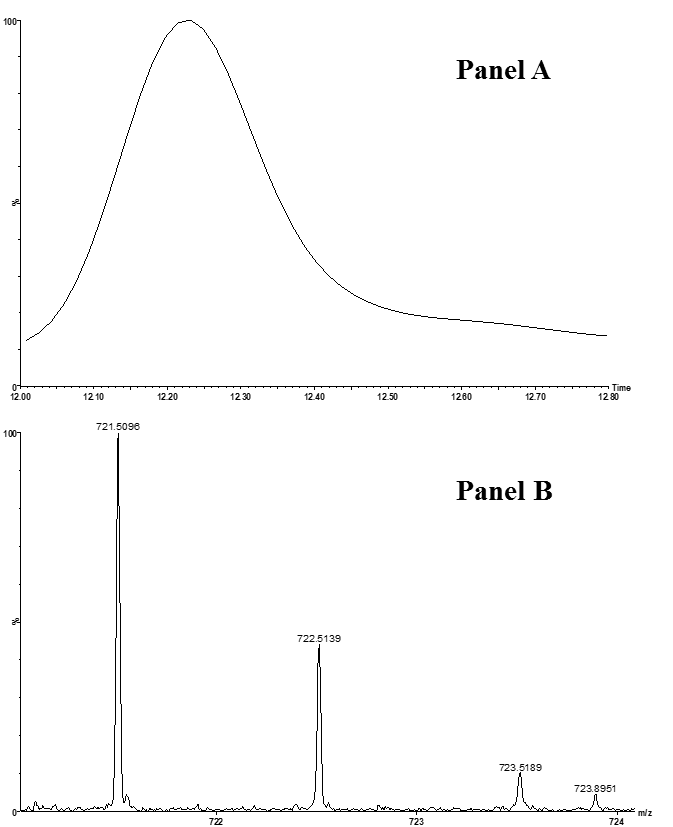
**

**Fig C.** Typical extracted ion chromatogram (Panel A) and relative spectrum under the peak (Panel B) showing the signal attributed to the phospholipid phosphatidyl-glycerol PG (C16:0/C18:0).

**
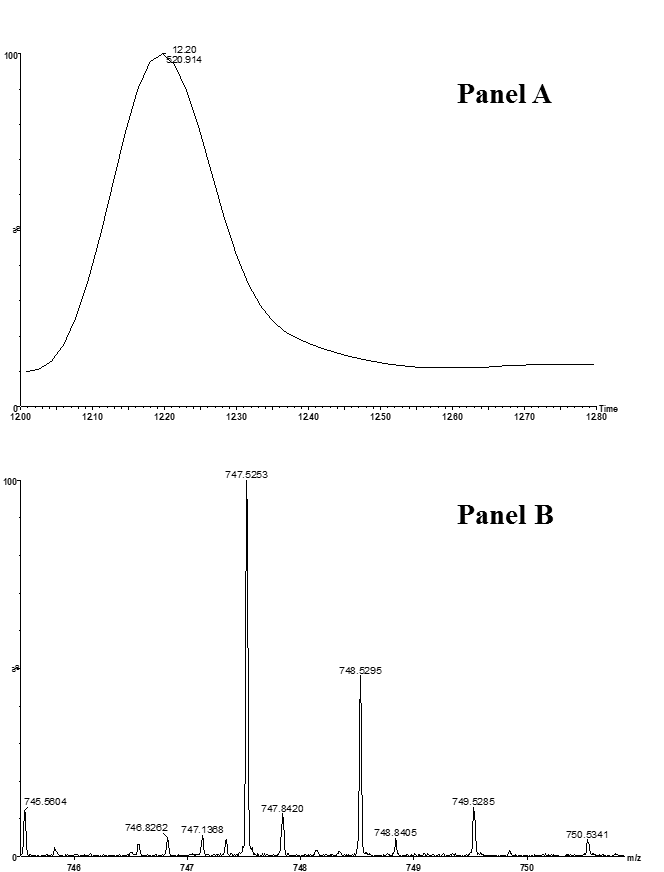
**

**Fig D.** Typical extracted ion chromatograms and relative spectrum under the peaks (Panel C) showing the signal attributed to the [M+3H]3+charge state of SP-C (monoisotopic m/z 1386.1222, Panel A) and to the [M+3H]3+charge state of its relative Met-Ox (monoisotopic m/z 1391.4562, Panel B).

**
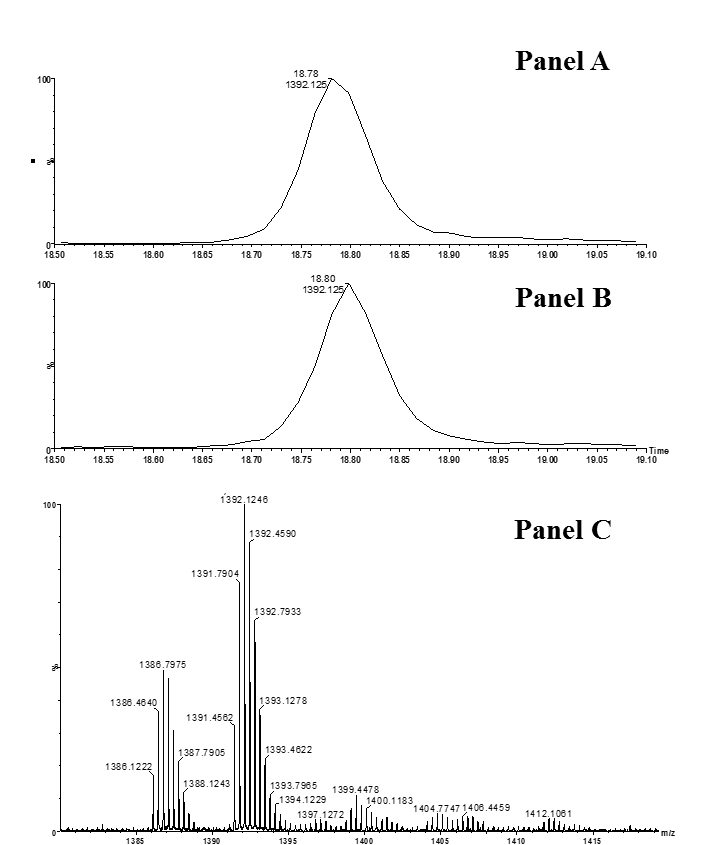
**

**Fig E.** Typical extracted ion chromatogram (Panel A) and relative spectrum under the peak (Panel B) showing the signal attributed to the [M+2H]^2+^ charge state of the proteotypic peptide IQAMIPK at m/z 421,77 obtained by trypsin digestion of the whole SpB intact protein.

**
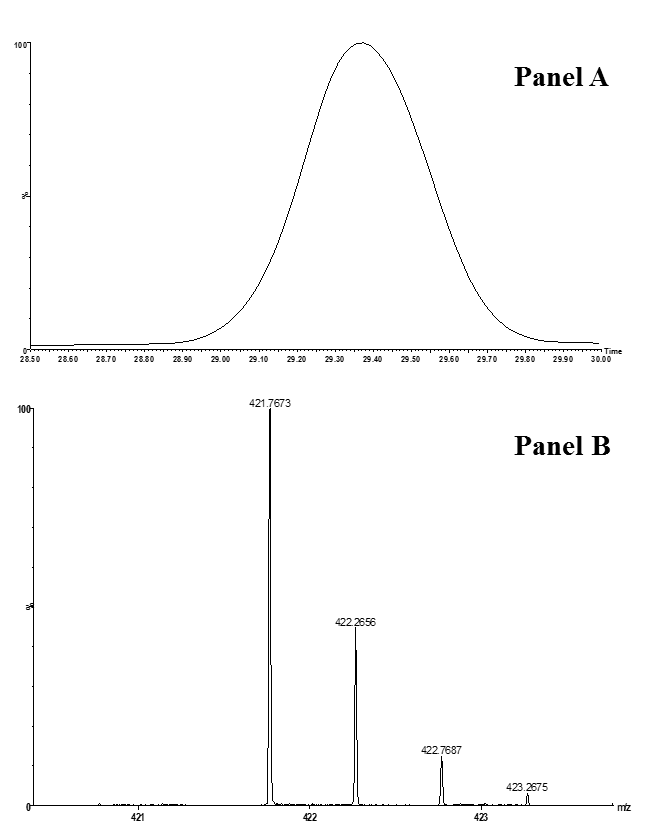
**

**Fig F.** Representative Pressure/Volume curves of surfactant-treated and untreated surfactant depleted rabbits. Pressure/Volume curves (P/V) performed *post mortem* in rabbits with respiratory failure induced by repeated broncho-alveolar lavages just managed with nCPAP (green curve), and in animals treated with a clinical dose of surfactant (Curosurf®, 200 mg/kg) delivered following the InSurE approach (surfactants instillation through the endotracheal tube followed by CPAP, red curve). The P/V curve was performed by progressively applying 5, 10, 15, 20, 25 and 30 ml of air-volume through a syringe and recording the pressure of the system at each volume point.


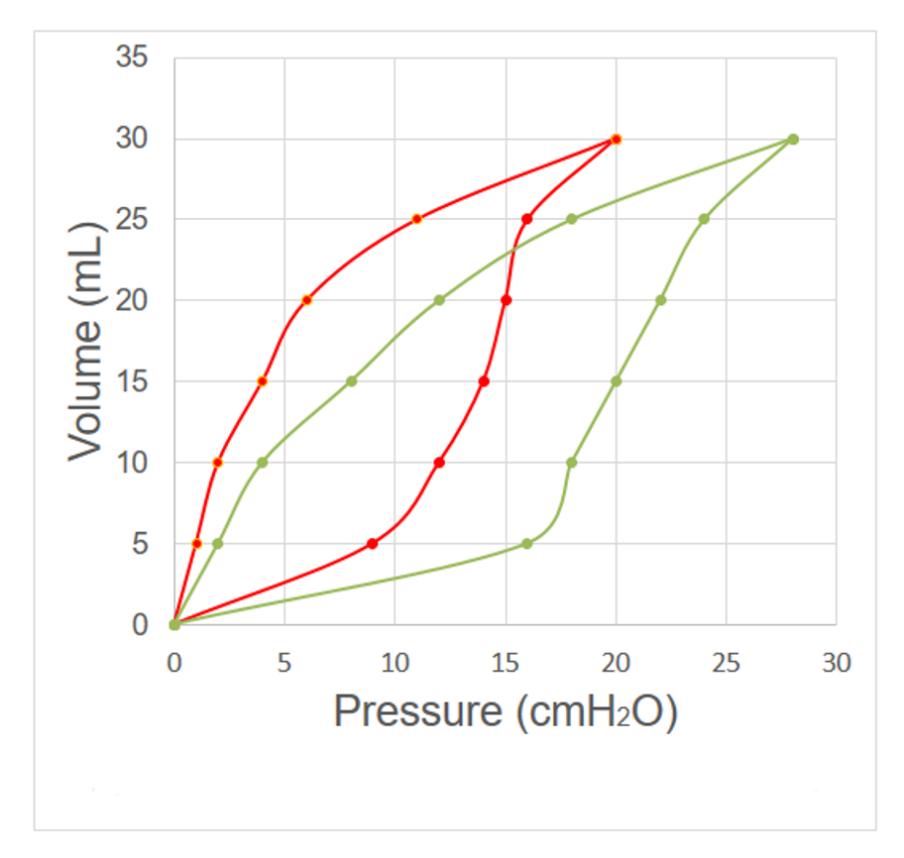


**Fig G.** Mean PaO_2_ in rabbits managed in CPAP after bronco-alveolar lavages. Mean PaO_2_ values in rabbits managed with nasal CPAP for 180 min that underwent at least 5 broncho-alveolar lavages (BALs, black squares), in rabbits managed with nasal CPAP for 180 min that just underwent 3 BALs (blue triangles) and in rabbits that underwent at least 5 BALs (red circles) treated with 200 mg/kg of surfactant (Curosurf®) following the InsSurE approach (surfactants instillation through the endotracheal tube followed by CPAP) and further managed for 180 min in nasal CPAP. Arterial blood gases were measured right after the induction of the anesthesia, upon intubation (baseline, B), after inducing the lung injury by repeated BALs and placing the animals on CPAP (0 min), 15 and 30 min after the start of CPAP ventilation, and then, every 30 min until the end of the experiment. Mean ± SEM are shown.


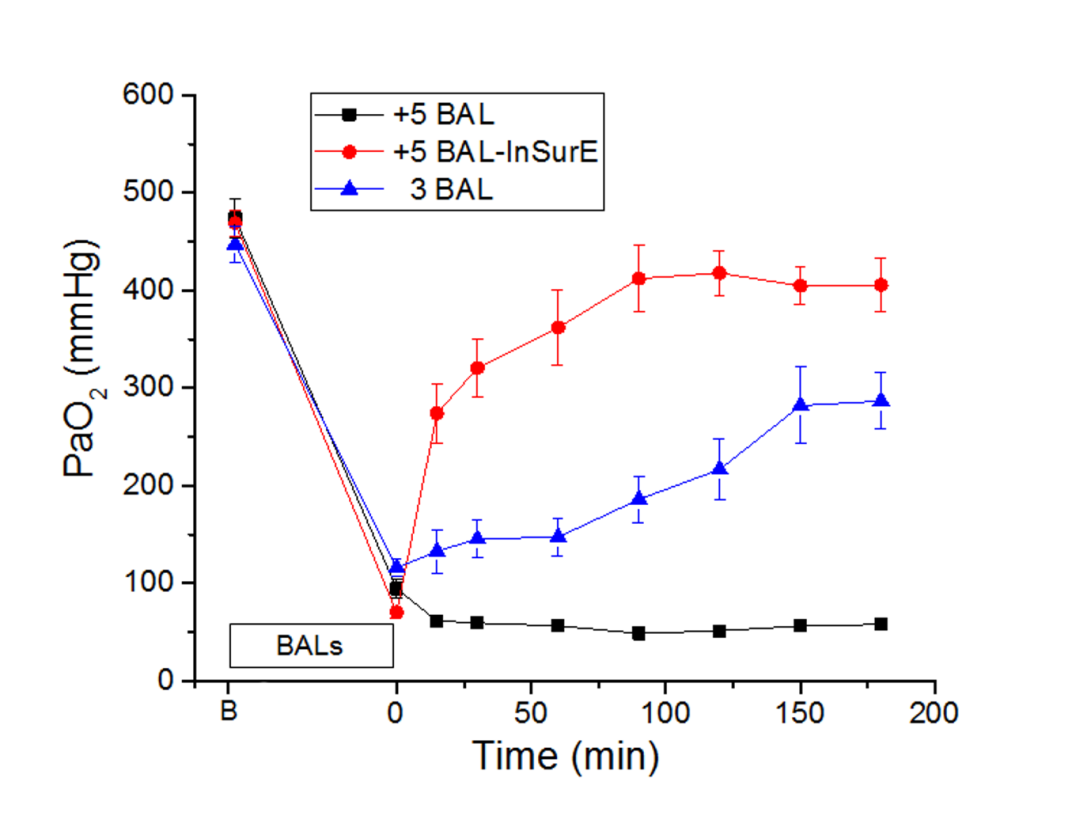

Supplement: S1 File — This file contains the following figures: Fig A. Typical extracted ion chromatogram (Panel A) and relative spectrum under the peak (Panel B) showing the signal attributed to the phospholipid phosphatidyl-choline (PC; C16:0/C16:0). Fig B. Typical extracted ion chromatogram (Panel A) and relative spectrum under the peak (Panel B) showing the signal attributed to the phospholipid phosphatidyl-glycerol (PG; C16:0/C16:0). Fig C. Typical extracted ion chromatogram (Panel A) and relative spectrum under the peak (Panel B) showing the signal attributed to the phospholipid phosphatidyl-glycerol PG (C16:0/C18:0). Fig D. Typical extracted ion chromatograms and relative spectrum under the peaks (Panel C) showing the signal attributed to the [M+3H]3+charge state of SP-C (monoisotopic m/z 1386.1222, Panel A) and to the [M+3H]3+charge state of its relative Met-Ox (monoisotopic m/z 1391.4562, Panel B). Fig E. Typical extracted ion chromatogram (Panel A) and relative spectrum under the peak (Panel B) showing the signal attributed to the [M+2H]2+ charge state of the proteotypic peptide IQAMIPK at m/z 421,77 obtained by trypsin digestion of the whole SpB intact protein. Fig F. Representative Pressure/Volume curves of surfactant-treated and untreated surfactant depleted rabbits. Pressure/Volume curves (P/V) performed post mortem in rabbits with respiratory failure induced by repeated broncho-alveolar lavages just managed with nCPAP (green curve), and in animals treated with a clinical dose of surfactant (Curosurf®, 200 mg/kg) delivered following the InSurE approach (surfactants instillation through the endotracheal tube followed by CPAP, red curve). The P/V curve was performed by progressively applying 5, 10, 15, 20, 25 and 30 ml of air-volume through a syringe and recording the pressure of the system at each volume point. Fig G. Mean PaO2 in rabbits managed in CPAP after bronco-alveolar lavages. Mean PaO2 values in rabbits managed with nasal CPAP for 180 min that underwent at least 5 b [file pone.0169190.s001.docx]
